# Tumour bone diseases and osteoporosis in cancer patients: pathophysiology, diagnosis, and therapy

**DOI:** 10.1054/bjoc.2000.1297

**Published:** 2000-07-03

**Authors:** Pauline Leonard

**Affiliations:** Clinical Research, UCL


*British Journal of Cancer* (2000) **83**(3), 418
© 2000 Cancer Research Campaign
doi: 10.1054/ bjoc.2000.1297, available online at http://www.idealibrary.com on IDEAL®

### Book Review

### Tumour bone diseases and osteoporosis in cancer patients: pathophysiology, diagnosis, and therapy

### Edited by Jean-Jacques body

The real joy in reading this book is that it truly combines the wealth of knowledge that already exists on normal bone pathophysiology and relates it relevantly to the biology of skeletal metastases so setting the scene for the application of both traditional and newer pharmacological treatments.

A very impressive collection of authors contributes to the 25 chapters, which are coherently arranged in six sections. It is written with the cancer physician in mind but the quality of the text, particularly the pathophysiology section, would capture the interest of any student at any level trying to comprehend the complexities of both normal and abnormal bone physiology. This book transforms the skeleton being viewed as a mystical if not somewhat ‘inert’ structure to that of an active organ whose function is intricately linked with the haemopoietic system.

Perhaps it is the pace of scientific discovery in the last 15 years, in particular as applied to the pathophysiology of bone, that has heightened interest in this area. Although very detailed the chapter on ‘Humoral hypercalcaemia of malignancy: the discovery of PTHrp’ quite elegantly takes the reader from the first principle of an observation, through a series of logical well-thought-out experiments using the technology as it became available, to the discovery of what Gutman in 1936 described as ‘complex factors as yet wholly obscure’.

The chapters generally follow on very nicely from one another, gradually building and expanding on the knowledge laid down in the preceding chapters. This has to be testament to the editorial skills of the editor Jean-Jacques Body who has successfully utilized the expert knowledge of the individual contributors without having too much repetition and overlap between chapters. Unfortunately, his task on the six chapters describing the pharmacology and increasing application of bisphosphonates in cancer management was made more difficult because of their obvious therapeutic success and potential. To date their use has revolutionized and simplified the management of hypercalcaemia in malignancy and been extended to not only the treatment of painful osteolytic metastases but also preventing morbidity associated with skeletal metastases. In addition, there is a body of emerging data on their role in preventing the development of skeletal and perhaps visceral metastases, not to mention their increasing use in osteoporosis associated with cancer, which makes them a very topical issue worthy of occupying a book on their own.

The chapter on orthopaedic management of skeletal metastases is particularly welcome as it is often relegated to a few lines in most other oncology textbooks. It is well written with good illustration and guidelines for management intervention.

I particularly enjoyed the overriding flavour of ‘food for thought’ topics contained in this book for, e.g. role of tumour markers in metastatic breast cancer involving the skeleton and use of HRT in patients put at high risk of developing osteoporosis by their cancer treatment. It is hard giving an up-to-the-minute account of all relevant data in emerging fields in a text book, but raising issues in this style and summarizing what is known so far gets around this problem somewhat.

I found this an extremely informative and challenging read. It is packed with so much information and data, particularly the early chapters, that several reads are required to gain most benefit. It is already a well-used text from my bookshelf.

*Dr Pauline Leonard MRCP*
*Clinical Research Fellow*
*UCL*

